# A Hypothesis: Metabolic Contributions to 16p11.2 Deletion Syndrome

**DOI:** 10.1002/bies.202400177

**Published:** 2024-12-29

**Authors:** Brandon Kar Meng Choo, Sarah Barnes, Hazel Sive

**Affiliations:** ^1^ Department of Biology Northeastern University Boston Massachusetts USA; ^2^ Health Sciences Department Sargent College of Health and Rehabilitation Sciences Boston University Boston Massachusetts USA

**Keywords:** 16p11.2 deletion syndrome, autism, body size, brain, copy number variation, metabolism, seizures

## Abstract

16p11.2 deletion syndrome is a severe genetic disorder associated with the deletion of 27 genes from a Copy Number Variant region on human chromosome 16. Symptoms associated include cognitive impairment, language and motor delay, epilepsy or seizures, psychiatric disorders, autism spectrum disorder (ASD), changes in head size and body weight, and dysmorphic features, with a crucial need to define genes and mechanisms responsible for symptomatology. In this review, we analyze the clinical associations and biological pathways of 16p11.2 locus genes and identify that a majority of 16p11.2 genes relate to metabolic processes. We present a hypothesis in which changes in the dosage of 16p11.2 metabolic genes contribute to pathology through direct or indirect alterations in pathways that include amino acids or proteins, DNA, RNA, catabolism, lipid, energy (carbohydrate). This hypothesis suggests that research into the specific roles of each metabolic gene will help identify useful therapeutic targets.

## Graphical abstract

16p11.2 Deletion syndrome is associated with brain, body, and behavioral symptoms. A majority of 16p11.2 genes relate to metabolism, involving amino acids/proteins, DNA, RNA, catabolism, lipids, and carbohydrates. Additional contributions include changes in cell structure, signaling, development, immune system, nervous system, and localization. We hypothesize that metabolic perturbation plays a major role in this prevalent syndrome.

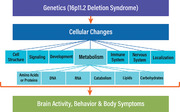

**Abbreviations**
ASD: autism spectrum disorder
CNV: copy number variation
iPSC: induced pluripotent stem cells
NMDA: N‐methyl‐D‐aspartate

## 1 Introduction

**FIGURE 1:**
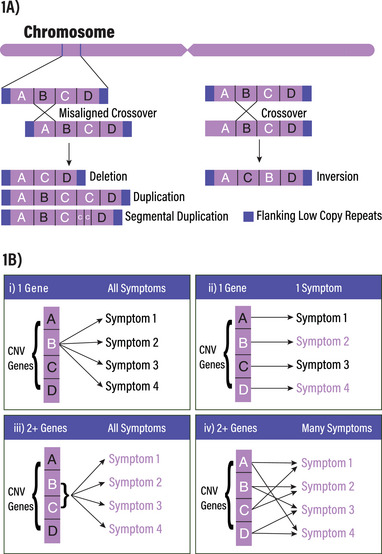
(A) Copy number variant (CNV) disorders involve deletion, duplication, segmental duplication, or inversion of DNA. (B) A change in gene copy number may be associated with symptoms in one of four predominant ways: (i) a single CNV gene is associated with all disease symptoms, (ii) each gene is responsible for a specific symptom, (iii) two or more CNV genes are responsible for all disease symptoms, or (iv) a composite set of interactions is associated with various symptoms. Figure 1B adapted from McCammon et al. [56].

Copy number variations (CNVs) occur when one or more chromosomal regions that are located at or near low copy repeats undergo deletion, duplication, or inversion. CNVs are prevalent across the human genome (with an estimated 4.8%–9.5% of the genome contributing [1]) and form through homologous and non‐homologous recombination (Figure 1A) [2]. The outcomes of genomic changes associated with CNV recombination are alterations in the copy number of genes in the CNV region, with possible effects on adjacent regions of the genome. CNV changes may be associated with phenotypes, disorders, or syndromes [3, 4].

### 1.1 Consequences of Changes in CNV Gene Copy Number

CNV regions often contain multiple protein‐coding genes, and a prevalent view is that a decrease or increase in gene dosage after CNV recombination is associated with phenotypic changes or symptoms. This association pertains to genes whose expression is not under compensatory control or where the activity of both chromosomal gene copies is needed for optimal function. There are several mechanisms by which “dosage‐sensitive” genes may be associated with phenotypes or symptoms when their copy number is altered (Figure 1B) [5]. The first is that *a single gene in the CNV is associated with all symptoms* (Figure 1Bi). For example, in Smith–Magenis syndrome, a microdeletion on the short arm of chromosome 17 (17p11.2) is connected to haploinsufficiency of the *RAI1* gene [6], as identified by phenotypic comparison of patients with this gene mutated or deleted [7], though the underlying molecular mechanisms are not well understood [8]. A second mechanism is that *each gene is associated with a specific symptom* (Figure 1Bii). For example, in DiGeorge syndrome (chromosome 22q11.2 microdeletion syndrome), whereby haploinsufficiency of the *TBX1* gene is strongly associated with immunodeficiency and cardiac structural abnormalities [9] while cognitive impairment is associated with *TANGO2*, another gene in the same region [10]. A third mechanism is that *two or more genes working together are associated with all symptoms* (Figure 1Biii). For example, in Cri du chat syndrome whereby deletion of the *SEMAF*, *CTNND2*, and *hTERT* genes in the chromosome 5p15.3–5p15.2 region is suggested to be collectively responsible for the phenotypes seen [11, 12]. A fourth mechanism is that *interaction of two or more genes is associated with many symptoms* (Figure 1Biv). For example, in Miller–Dieker syndrome (chromosome 17p13.3 deletion syndrome), microdeletion of the *PAFAH1B1* gene produces lissencephaly associated with the syndrome. However, a deletion that also affects the *YWHAE* gene in the same region markedly worsens the severity of lissencephaly [13], suggesting interaction between these genes. Composites of these mechanisms are also feasible.

### 1.2 CNVs May Have Effects on Other Genomic Regions

Connection between copy number variation and phenotypes may involve processes outside gene dosage. One mechanism could involve fusion of different genes or their regulatory sequences due to CNV recombination, such that a new gene product is produced, or the regulation of a gene is altered [4]. A second mechanism is that a CNV change could alter the activity of genes outside the CNV, through chromatin‐mediated alterations [14, 15]. A third mechanism is that deletion of a region on one chromosome in the CNV region unmasks a recessive allele or functional polymorphism on the other chromosome and leads to a new phenotype [16]. A fourth mechanism is an interruption of “transvection,” communication between two paired genes on homologous chromosomes, due to the deletion of regulatory elements [4].

## 2 The 16p11.2 Region: A Syndrome‐Linked CNV

The 16p11.2 region of the human genome undergoes frequent copy number variation, and these changes are associated with severe symptoms, variably present in affected people. Symptoms include cognitive impairment, language and motor delay, epilepsy or seizures, psychiatric disorders, and autism spectrum disorder (ASD), changes in head size and body weight, and dysmorphic features. Our interest in this CNV region has been to understand the key genes involved and to identify personalized therapies that target underlying causes. As we will explore in Section 3, analysis of the region has led to a new hypothesis that metabolic changes may contribute to associated symptoms.

### 2.1 The 16p11.2 CNV Undergoes Deletion and Duplication With Symptomatic Outcomes

The 16p11.2 region is located on the short arm of human chromosome 16 and is found in the 11th band and 2nd sub‐band after Giemsa staining. This region includes 27 protein‐coding genes and is flanked by approximately 147 kilobase pair segmental duplications that are more than 99% identical [17, 18]. These flanking regions are believed to promote 16p11.2 deletion and duplication, if they become misaligned during non‐allelic homologous recombination [17].

The two major copy number variations associated with the 16p11.2 region are duplication and deletion, occurring at similar frequencies with an average prevalence of 0.044% (∼1 in 2500 people) with 16p11.2 deletion, and 0.036% (∼1 in 2700 people) with duplication [19]. Symptoms are associated with both 16p11.2 deletion or duplication, although the deletion phenotype is more severe [19, 20, 21, 22]. 16p11.2 deletion syndrome (16pdel) symptoms include cognitive impairment, language and motor delay, epilepsy or seizures, psychiatric disorders, and ASD, macrocephaly, obesity and dysmorphic features [23]. Notably, 16p11.2 deletion is the most prevalent CNV associated with ASD, accounting for almost 1% of all cases [17, 24, 25].

Interestingly, when symptoms associated with a deletion in the 16p11.2 region are compared to those from the duplication, opposing “mirror” phenotypes can sometimes be seen. For example, deletion is associated with macrocephaly, increased brain volume and obesity whereas duplication is associated with microcephaly, decreased brain volume and being underweight [26]. This reciprocity is not seen in other symptoms including ASD, Attention Deficit Hyperactivity Disorder (ADHD) [21] as well as motor delay and seizures [27], which are associated with both deletion and duplication. Since 16pdel symptoms are significantly more severe than those associated with 16p11.2 duplication, we will focus on consideration of the deletion syndrome for the remainder of this review.

### 2.2 What Genes Contribute to 16pdel Phenotypes?

The 16p11.2 locus includes 27 genes in the core (non‐repeat) region (in order along the chromosome: *SPN*, *QPRT*, *C16orf54*, *ZG16*, *KIF22*, *MAZ*, *PRRT2*, *PAGR1*, *MVP*, *CDIPT*, *SEZ6L2*, *ASPHD1*, *KCTD13*, *TMEM219*, *TAOK2*, *HIRIP3*, *INO80E*, *DOC2A*, *C16orf92*, *TLCD3B*, *ALDOA*, *PPP4C*, *TBX6*, *YPEL3*, *GDPD3*, *MAPK3*, and *CORO1A*). This large gene set encodes many kinds of protein, including those assigned as transcription factors (e.g., *TBX6*), signaling molecules (e.g., MAPK3), enzymes (e.g., *ALDOA*), mitotic regulators (e.g., *KIF22*), chromatin modifiers (e.g., INO80E), synaptic regulators (e.g., DOC2A), as well as those of unknown or less defined activity (Table 1). The challenge is to link specific 16p11.2 genes with corresponding phenotypes or symptoms. The need is to know which 16p11.2 genes are associated with a single phenotype, and which may work together to connect with a phenotype (Figure 1B). This knowledge is crucial to define informed therapeutic approaches that address mechanisms underlying each symptom. The complexity involved is daunting, but progress is being made, and we present new analyses that add to understanding.

**TABLE 1:** Genes of the 16p11.2 locus region and corresponding or predicted protein functions. Gene order represents order along chromosome 16. Assignments from human or mouse NCBI databases, with corresponding human or mouse NCBI IDs.

Gene	Full gene name	NCBI human or mouse ID	Corresponding or predicted protein function
*SPN*	Sialophorin	6693/20737	Antigen‐specific activation of T cells
*QPRT*	Quinolinate phosphoribosyltransferase	23475/67375	Enzyme, catabolism of quinolinate
*C16orf54*	Chromosome 16 open reading frame 54	283897/101602	Predicted integral membrane protein
*ZG16*	Zymogen granule protein 16	653808/69036	Predicted carbohydrate, peptidoglycan binding, protein transport
*KIF22*	Kinesin family member 22	3835/110033	Chromosomal movement during cell division
*MAZ*	MYC associated zinc finger protein	4150/17188	Transcription co‐activator
*PRRT2*	Proline rich transmembrane protein 2	112476/69017	Transmembrane protein, predicted synaptic regulation
*PAGR1*	PAXIP1 associated glutamate rich protein 1	79447/67278	Transcriptional co‐activator, epigenetic regulation
*MVP*	Major vault protein	9961/78388	Major component of the vault complex
*CDIPT*	CDP‐diacylglycerol–inositol 3‐phosphatidyltransferase	10423/52858	Enzyme, phosphatidylinositol synthesis
*SEZ6L2*	Seizure related 6 homolog like 2	26470/233878	Seizure‐related protein
*ASPHD1*	Aspartate beta‐hydroxylase domain containing 1	253982/233879	Predicted to enable dioxygenase activity, peptidyl‐amino acid modification
*KCTD13*	Potassium channel tetramerization domain containing 13	253980/233877	Part of the Cul3‐RING ubiquitin ligase complex
*TMEM219*	Transmembrane protein 219	124446/68742	Apoptotic regulator
*TAOK2*	TAO kinase 2	9344/381921	MAPK kinase signaling pathway, serine/threonine kinase
*HIRIP3*	HIRA interacting protein 3	8479/233876	Chromatin and histone metabolism
*INO80E*	INO80 complex subunit E	283899/233875	DNA recombination, DNA repair, chromatin remodeling
*DOC2A*	Double C2 domain alpha	8448/13446	Calcium dependent neurotransmitter release
*C16orf92*	Chromosome 16 open reading frame 92	146378/78118	Sperm‐oocyte fusion, membrane protein
*TLCD3B*	TLC domain containing 3B	83723/68952	Ceramide synthase modulator
*ALDOA*	Aldolase, fructose‐bisphosphate A	226/11674	Glycolytic enzyme
*PPP4C*	Protein phosphatase 4 catalytic subunit	5531/56420	Enzyme, Protein serine/threonine phosphatase
*TBX6*	T‐box transcription factor 6	6911/21389	Transcription factor
*YPEL3*	Yippee like 3	83719/66090	Regulates cellular senescence
*GDPD3*	Glycerophosphodiester Phosphodiesterase domain containing 3	79153/68616	Enzyme, lysophospholipase, phosphoric diester hydrolase
*MAPK3*	Mitogen‐activated protein kinase 3	5595/26417	MAPK kinase signaling pathway, serine/threonine kinase
*CORO1A*	Coronin 1A	11151/12721	WD repeat protein, actin‐binding

Changes in 16p11.2 gene dosage after deletion of one chromosomal copy have been associated with a decrease in total expression from each gene [28, 29, 30]. These data are consistent with the discussion in Section 1.1, whereby 16pdel phenotypes are a direct consequence of changes in gene dosage. It has also been suggested that 16p11.2 deletion or duplication alters surrounding chromatin and gene expression and that this contributes to the 16p11.2 CNV phenotypes. Consistently there is a differential expression of some genes flanking the deletion, including those involved in chromatin modification, methylation, and transcription [14], some of which have been associated with ASD or other psychiatric diseases [14, 15]. Furthermore, the variability in symptoms observed in people with 16p11.2 deletion likely indicates contributions of other genomic regions to specific symptomatology. However, based on dosage‐dependent gene expression, and data connecting most 16p11.2 genes in the core interval with symptoms (as discussed in the following sections), it is likely that much 16pdel pathophysiology can be accounted for by changes in dosage of the 27 genes in the core interval.

#### 2.2.1 Disease Associations of 16p11.2 Region Genes

The first consideration around which 16p11.2 genes (Table 1) drive specific symptoms focuses on genetic correlations of 16p11.2 human gene variants (where a “variant” refers to slight differences in DNA sequence of a specific gene across the population) and specific disorders. From Genome Wide Association Studies (GWAS) across all DNA regions, or exome sequencing (of protein‐coding regions), the literature identified only five 16p11.2 genes where a single gene variant is associated with a specific disorder as shown in Table S1. Through GWAS analysis, *TAOK2* putative loss of function variants were identified in families with ASD [31]. A putative loss of function variant of *CORO1A* was connected to severe combined immunodeficiency syndrome [32]. Using whole exome sequencing, variants of *PRRT2* were found in patients with epilepsy/seizures and/or paroxysmal kinesigenic dyskinesia [33, 34, 35]. Multiple studies have focused on *TBX6*, and exome sequencing has identified apparent loss of function variants in patients with congenital anomalies, including vertebral malformations [36], scoliosis [37], urinary tract and kidney anomalies [36] as well as female reproductive system anomalies [38]. A variant in regulatory sequences of *TLCD3B* was identified among a large data set associated with ASD [39]. Since only five genes show single variant‐phenotype connections, the data indicate that most 16p11.2 genes do not singly contribute to one or multiple symptoms (Figure 1Bi,ii) and that combinations of genes are most likely to regulate all 16pdel symptoms (Figure 1B iii,iv).

**FIGURE 2:**
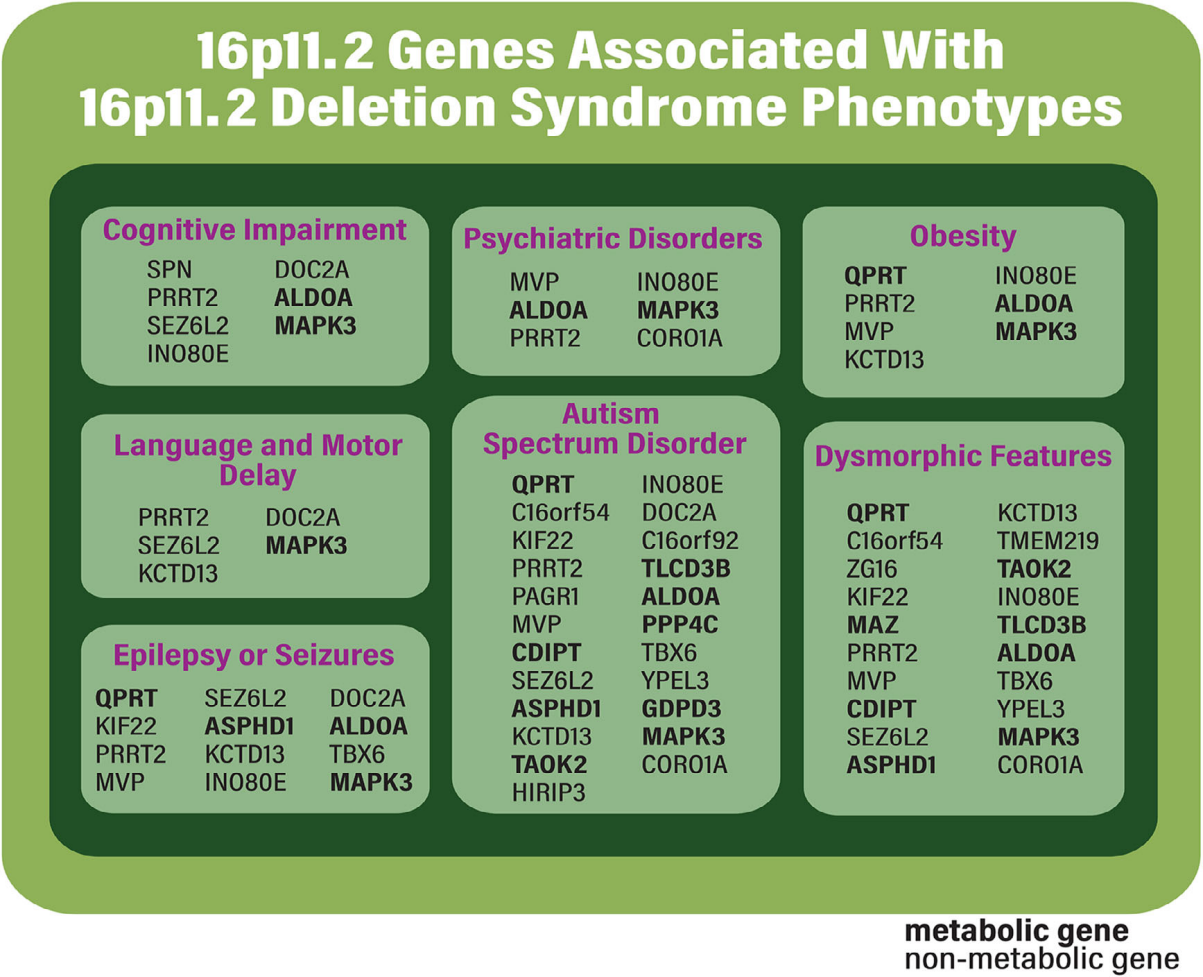
16p11.2 locus genes and associated diseases from the literature and Malacards (Version 5.2). Most information on Malacards is generated using two methods, the first method to search manually curated databases such as OMIM, the second to search text‐mining sources such as Novoseek, with a link to the evidence‐providing publications [41]. Associations from both these methods are integrated to show disorders that match the search term. Bolded genes signify metabolic genes.

To help assess the combinatorial gene interactions likely to direct symptomatology of 16pdel, we searched the MalaCards database that integrates gene‐disease associations using a broad spread of data ranging from human genetic analysis to experimental model analyses [40]. As shown in Figure 2, most of the 27 core 16p11.2 genes were indicated as potentially associated with ASD (23 genes), followed by dysmorphic features (20 genes), epilepsy or seizures (12 genes), cognitive impairment and obesity (seven genes each), psychiatric disorders (six genes), language and motor delay (five genes). Interestingly, 11 of these genes encode metabolic functions (**bolded**). This analysis indicates that multiple 16p11.2 genes can be associated with each 16pdel phenotype, underscoring that most symptoms are likely to be controlled by combinations of genes (Figure 1Biii,iv), which aligns with previous findings [41]. It should be noted that while 16pdel is highly penetrant, the combination of phenotypes in each affected individual is variable [19].

#### 2.2.2 Experimental Analysis Shows 16p11.2 Genes Are Active and Interactive

One way by which researchers are exploring how 16p11.2 interval genes are linked to deletion syndrome symptoms is through experimental analysis in model systems. Systems analyzed include tissue culture cells (from human or non‐human sources), and animal models such as fruit flies, zebrafish, rats, and mice, using genetic or molecular loss of function analyses. The data indicates that gene combinations are responsible for 16pdel genes can functionally interact. We note that resulting phenotypes observed in non‐human systems may not precisely recapitulate human phenotypes, and we previously used the term “tool” (rather than “model”) to indicate that non‐human systems provide insight into gene function, regardless of the phenotype [23, 42].

In species examined, most human 16p11.2 locus genes have a non‐human homolog [28, 43–45]. The 16p11.2 interval has a syntenic (matching) region in primates [46] and rodents [28, 45], with the set of genes contiguously localized. In zebrafish [43] and fruit flies (*Drosophila melanogaster*) [44], homologous genes are distributed across one or more chromosomes. In mice, loss of one copy of the homologous 16p11.2 interval is associated with severe outcomes including reduced weight, impaired adipogenesis, hyperactivity, repetitive behaviors, and memory deficits [28], many similar to those seen in people [23]. Effects of 16p11.2 deletion can be seen in cultured cells, and human‐induced pluripotential cell lines (iPSCs) from people affected show changes in activity or metabolism [47, 48].

Non‐human studies focusing on function of single or pairs of genes have been reported for all but four (*ZG16*, *PRRT2*, *TMEM219*, and *C16orf92*) genes. These studies generally include knockout (genetic loss of both copies), haploinsufficiency (genetic loss of one functional copy), or knockdown (using antisense RNA, oligonucleotides, or other means) (Table S2).

With regard to ASD, changes in human brain morphology are associated with spectrum disorders [49, 50], and animal studies identified a change in brain morphology, such the brain ventricles of zebrafish larvae [43], aberrant targeting of presynaptic axons to postsynaptic targets [51] or neuronal morphology [52]. ASD includes effects on social behavior, and in mice, loss of function of *Taok2* was associated with social impairment [31]. With regard to dysmorphic features, meaning body structure anomalies, several genes (*SPN, C16orf54, SEZ6L2, ASPHD1, TAOK2, INO80E*, and *TLCD3B*) contribute to the development of craniofacial features in humans, mice, rats, and zebrafish [53]. *TBX6* is associated with congenital anomalies of the kidney and urinary tract, in both humans and mice [36]. With regard to epilepsy or seizures, there has been the use of acute seizure models [5] and/or chronic seizure models [44, 54, 55]. In zebrafish, *tlcd3b* was associated with increased seizure susceptibility when both gene copies are knocked out (homozygous), but not when only one is (heterozygous) [5]. In humans and rats, abnormal protein expression of DOC2A was implicated in epilepsy [55], while in *Drosophila*, homologs of the human genes *MAPK3* and *PPP4C* were associated with a higher propensity for seizures [44], with *Kctd13* associated in both mouse models and *Drosophila* [44, 54]. With regard to anxiety, *Kctd13* and *Taok2* loss of function in mice promoted anxiety‐like behaviors. With regard to learning and memory, *Kctd13* [56, 57] or *Taok2* [31] loss of function impaired these abilities in mice. With regard to body size, while Mapk3 can change obesity‐related parameters such as adipogenesis and adiposity in mice, there is conflicting evidence on whether *Mapk3* loss of function increases [58] or decreases obesity [59, 60]. *Mvp* loss of function increased obesity in mice in the context of a high‐fat diet [61] and knockout of *Tlcd3b* was associated with increased body size in zebrafish [5]. These studies add to the understanding that genes in the 16p11.2 interval are highly active, supporting a complex set of contributions to deletion syndrome phenotypes.

**FIGURE 3:**
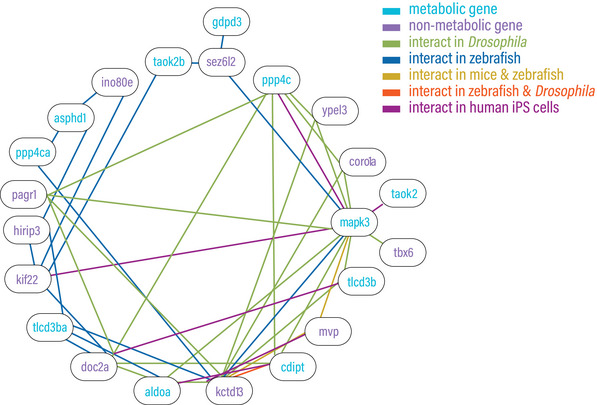
Multiple 16p11.2 deletion syndrome genes, with both metabolic and non‐metabolic functions show pairwise interactions, from studies in zebrafish, Drosophila, mice, and human tissue culture cells.

Based on data supporting multigenic contributions to 16p11.2 deletion symptomatology, work from our group [5, 43, 48] as well as others [53, 62] in zebrafish, in *Drosophila* [44], in mice [63], humans [25], and human iPSC [64] shows that a significant number of gene pairs in the 16p11.2 region can functionally interact (Figure 3). For example, *doc2a* and *tlcd3b* double heterozygote (knockdown) zebrafish were hyperactive and susceptible to seizures and showed an increase in body length and head size, relative to single gene heterozygotes [5]. Computational analysis integrating genetic and gene expression data suggests that pairwise interaction of 16p11.2 genes better explained symptomatic differences between affected people among five chosen traits (bipolar disorder, schizophrenia, BMI, and IQ) [25]. *DOC2A* plus *TLCD3B*, was a top associated pair for BMI (obesity) and IQ (cognitive impairment), consistent with our data in zebrafish [5], *CDIPT* plus *ALDOA* the top pair for BMI, and *KCTD13* plus *MVP* for IQ [25].

These experimental analyses indicate that the majority of 16p11.2 genes can be associated with phenotypes in knockdown or knockout studies of experimental models. Some of these phenotypes appear similar or connected to human symptoms, including brain and body phenotypes. The interactions documented to date are pairwise only, and many additional genetic interactions of 16p11.2 locus genes likely remain to be discovered.

#### 2.2.3 Biological Pathways of Genes in the 16p11.2 Region

To understand further how each 16p11.2 gene may contribute to deletion syndrome symptoms, the array of biological pathways in which each is involved needs to be clarified. Therefore, for each of the 27 core 16p11.2 genes, contributions to biological processes were examined by assigning gene ontology (GO) terms, that suggest functional contributions based on the literature.

**FIGURE 4:**
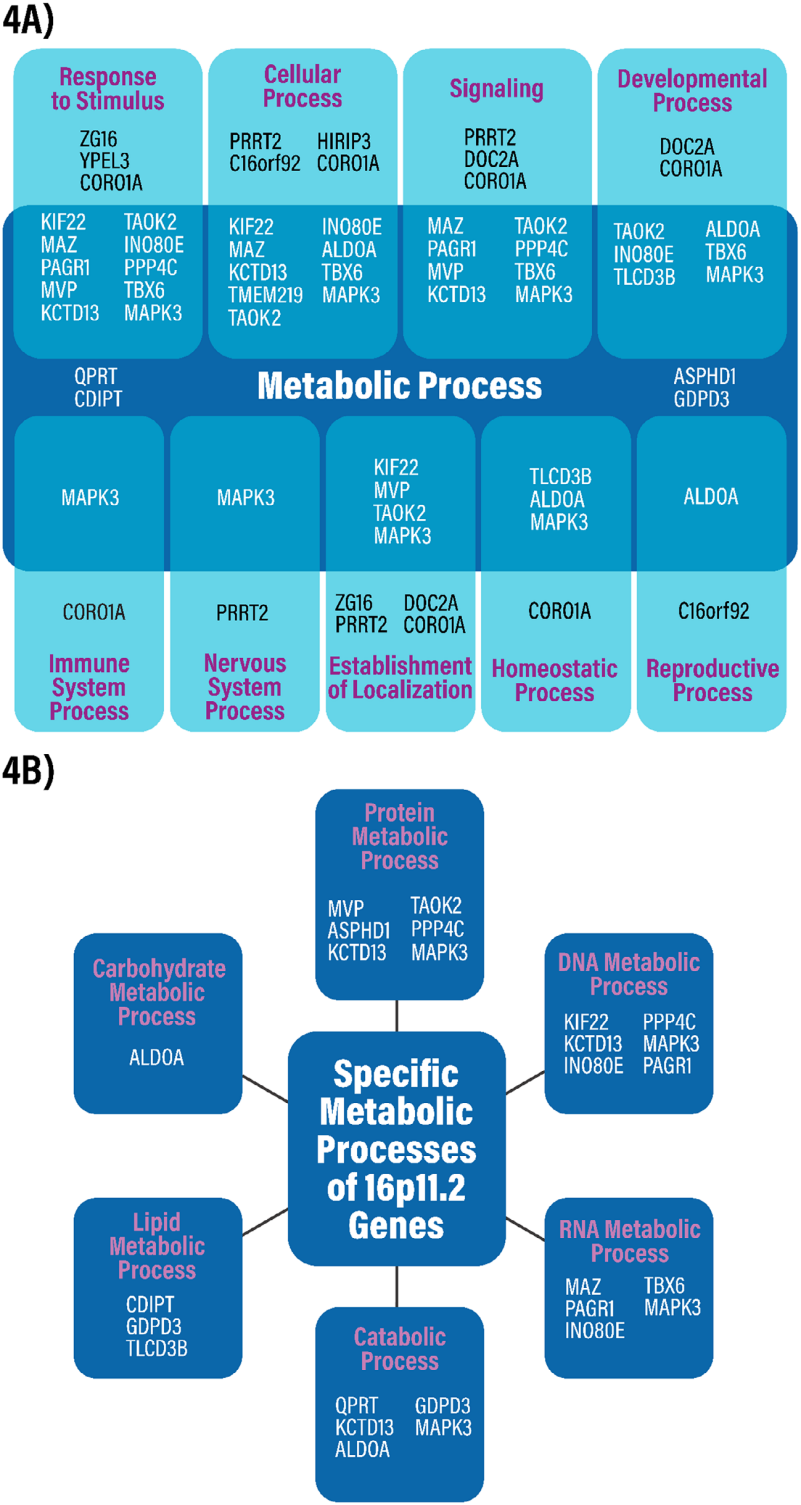
(A) Gene ontology annotations of 16p11.2 deletion syndrome genes with overlap signifying genes which have Metabolic Processes annotations in addition to another annotation. (B) Diagram showing the specific individual gene ontology metabolic annotations that make up the general term Metabolic Processes in (A). The Generic Gene Ontology (GO) Term Mapper was used to annotate the genes. The GO slim file used was the AGR subset dated 2024‐06‐17.

The results (Figure 4A) indicate that genes are involved in “Metabolic Process,” “Response to Stimulus,” “Cellular Process,” “Signaling,” “Developmental Process,” and “Establishment of Localization.” In contrast, relatively fewer genes were involved in “Immune System Process,” “Nervous System Process,” “Homeostatic Process,” and “Reproductive Process.” Notably, of the 27 genes (Table 1), “Metabolic Process” had the highest number (16) of annotations (*QPRT, KIF22, MAZ, PAGR1, MVP, CDIPT, ASPHD1, KCTD13, TAOK2, INO80E, TLCD3B, ALDOA, PPP4C, TBX6, GDPD3*, *and MAPK3*). Most genes were indicated as part of several processes, but four genes (*QPRT*, *CDIPT*, *ASPHD1*, and *GDPD3*) were annotated as exclusively involved in “Metabolic Process.”

The finding (Figure 4A) that most 16p11.2 locus genes are connected to metabolic processes leads us to hypothesize that dysfunctional metabolic mechanisms may underlie a substantial portion of phenotypes associated with 16pdel. We define “metabolism” as the set of chemical reactions required for cellular function, including the synthesis, degradation, modification, and re‐distribution of compounds contributing to homeostasis. We define “metabolic genes” as those whose product/s participate in the regulation of metabolism, including enzymes and their modulators. We stratified the distinct aspects of metabolism connected to each gene (Figure 4B), where the top categories are protein metabolism, DNA metabolism, RNA metabolism, catabolic processes, lipid metabolism, and carbohydrate metabolism.

## 3 A Cohort of Metabolic Regulatory Genes in the 16p11.2 Region That Connect With 16pdel Phenotypes

Given the significant number of 16p11.2 genes assigned to metabolism‐related biological pathways (Figure 4), and symptoms (Figure 2), and aggregated in Table S4, we examined the possible contributions of metabolic genes to phenotypes seen in 16pdel. Of the sixteen 16p11.2 locus genes identified as contributing to metabolism, some do so indirectly, for example, *MVP* [61]. We focus on ten genes in the 16p11.2 region identified as enzymes or modulators and reported to control cellular biochemistry directly, and how each might contribute to certain 16pdel symptoms (Figure 2). These genes are *QPRT*, *MAZ*, *CDIPT*, *ASPHD1*, *TAOK2*, *TLCD3B*, *ALDOA*, *PPP4C*, *GDPD3*, and *MAPK3*. Although *ASPDH1* is annotated as metabolic, and includes an Aspartate Dehydrogenase domain, no enzymatic function has been assigned to this gene (Alliance of Genome Resources, Nov 2024).

### 3.1 Amino Acids or Protein Metabolism—ASPHD1, TAOK2, PPP4C, MAPK3

Aspartate beta‐Hydroxylase Domain containing 1 or ASPHD1 (EC 1.14.11) is a protein predicted to have aspartate dehydrogenase activity, and hence is ascribed as metabolic. Knockdown of the zebrafish gene resulted in defective brain morphology, albeit weak phenotypes, as well as tail and muscle segment defects [43]. However, we did not ascribe ASPHD1 to any 16pdel phenotypes as its function is not well defined.

Thousand and One Amino Acid Protein Kinase 2 or TAOK2 (EC 2.7.11.1) is a serine/threonine kinase in the MAP kinase pathway that is involved in translation elongation [65]. *Taok2* loss of function is associated with increased brain size, and brain structural and synaptic defects in mice [31, 63, 66]. Consistently, knockdown of the zebrafish homolog reduced brain ventricle size and altered the midbrain‐hindbrain boundary [43]. *Taok2* knockout mice showed impairment in cognition, anxiety, and social interaction [31]. Knocking down the *Drosophila* analog of *TAOK2* increased triacylglyceride levels through the Hippo signaling pathway associated with adiposity [67]. These findings may explain TAOK2 association with the 16pdel symptoms of ASD, obesity, and dysmorphic features.

Protein Phosphatase 4 Catalytic Subunit or PPP4C (EC 3.1.3.16) is a serine/threonine phosphatase and contributes to organelle assembly, cellular signaling, and nuclear functions [68]. Knocking down *Drosophila PPP4C* resulted in wing defects [44], while in mice, loss of *Ppp4c* function produced neuroanatomical defects and reduced brain size [63]. Knockdown of the zebrafish homolog resulted in defective brain morphology and axon tracts, as well as movement defects [43]. These findings may suggest an association of PPP4C with ASD in 16pdel.

Mitogen‐activated Protein Kinase 3 (MAPK3) or ERK1 (EC 2.7.11.24), is a serine/threonine protein kinase in the Ras‐Raf‐MEK‐ERK signaling pathway that regulates cell growth, division, death, and other cellular processes [69]. MAPK3 homologs regulate axonal targeting [51], and may connect to hyperproliferation of neural precursor cells suggested to occur in ASD [70] and macrocephaly [62, 70]. This hyperproliferation is accompanied by a premature depletion of progenitor pools, and cortical cytoarchitecture alteration seen in 16pdel mice models [71]. Mice with *Mapk3* loss of function displayed anxiety‐like behaviors [71] as well as impaired memory and cognition [57, 71], possibly through indirect effects [72]. *MAPK3* loss of function is associated with locomotion dysfunction in *Drosophila* [44] and zebrafish [43] as well as increased seizure susceptibility in *Drosophila* [44]. These findings implicate MAPK3 association with the 16pdel symptoms of cognitive impairment, psychiatric disorders, motor delay, ASD, as well as dysmorphic features.

### 3.2 DNA Metabolism—PPP4C, MAPK3

The two relevant genes, PPP4C and MAPK3 are discussed in Section 3.1.

### 3.3 RNA Metabolism—MAZ, MAPK3

MYC Associated Zinc Finger Protein or MAZ may play a role in DNA binding activity and transcription (Alliance of Genome Resources, Nov 2024). Knockdown of the gene in zebrafish resulted in defective brain and eye morphology and spontaneous movement defects [43]. Abnormal ocular development was also found in humans with *MAZ* variants [73].These findings regarding MAZ could explain its association with the 16pdel symptoms of motor delay and dysmorphic features. *MAPK3* was discussed in Section 3.1.

### 3.4 Catabolism—QPRT

Quinolinate phosphoribosyl transferase or QPRT (EC 2.4.2.19), is an enzyme involved in the catabolism of quinolinate [74], which is believed to be neuroexcitatory [75]. *QPRT* loss of function leads to increased quinolinate, overactivation of the N‐methyl‐D‐aspartate (NMDA) receptors, and is associated with neurodegenerative disorders and neuronal cell death [76] and seizures [77]. High levels of quinolinate in the striatum of young rats inhibit energy metabolism by compromising oxidative phosphorylation, the citric acid cycle and cellular energy transfer [78]. Quinolinate may increase oxidative stress by downregulating the activity of certain endogenous antioxidants [79], resulting in the neuronal oxidative damage seen in neurodegenerative diseases [80]. The primary ligand for NMDA receptors is glutamate and quinolinate may overactivate the glutamatergic system by inhibiting vesicular glutamate uptake and enhancing synaptosomal glutamate release [81]. These findings could explain QPRT association with the 16pdel symptoms of obesity, ASD, epilepsy or seizures.

### 3.5 Lipid Metabolism—CDIPT, TLCD3B, GDPD3

CDP‐Diacylglycerol–Inositol 3‐Phosphatidyltransferase or CDIPT (EC 2.7.8.11), is an enzyme that regulates the synthesis of intracellular phosphatidylinositol from myo‐inositol and CDP‐diacylglycerol [82], a key step in the phosphatidylinositol cycle [83, 84]. Knocking down the *CDIPT* homolog in *Drosophila* resulted in neuromuscular junction alterations [44], while in zebrafish, the homozygous *cdipt* knockout was larval lethal [85], with fin defects, muscle and motor defects, and altered liver metabolism [86], while knockdown showed reduced brain ventricle size and a less sharply defined midbrain‐hindbrain boundary [43]. These findings are consistent with a role for CDIPT in the 16pdel symptoms of ASD and dysmorphic features.

TLC Domain Containing 3B or TLCD3B, also known as FAM57B, is a modulator of ceramide synthase activity [48]. The synthesis of sphingomyelin produces diacylglycerol, which participates in the phosphatidylinositol cycle, in cell membranes and as a second messenger [87]. Work from our group in zebrafish and human cells showed that a lack of *TLCD3B* changes the composition and levels of sphingolipids and glycerolipids associated with cell membranes [48, 88]. Unlike a previous publication [89] suggesting that TLCD3B is a ceramide synthase, we showed that TLCD3B is a ceramide synthase modulator, that may increase or decrease the function of specific ceramide synthases. *Tlcd3b* may play a role in obesity as ceramides inhibit adipogenesis, through interaction with the obesity‐related gene *Pparγ* [89]. Research from our group demonstrated that zebrafish *tlcd3b* homolog mutants displayed an increased susceptibility to seizures, increased head size [5], defective brain morphology and spontaneous movement defects [43]. These findings indicate how TLCD3B could be associated with the 16pdel symptoms of ASD and obesity.

Glycerophosphodiester Phosphodiesterase Domain Containing 3 or GDPD3 (EC 3.1.4.46), is an enzyme that hydrolyzes lysophospholipids into lysophosphatidic acids that can affect other phospholipids [90]. Loss of function of this gene is associated with morphological changes and movement deficiency in zebrafish larvae [43]. These data suggest that GDPD3 functions may contribute to the 16pdel symptom of motor delay.

### 3.6 Carbohydrate Metabolism—ALDOA

Aldolase, Fructose‐Bisphosphate A or ALDOA (EC 4.1.2.13), is an enzyme in the energy‐producing process of glycolysis [91]. Glycolytic G enzymes such as *ALDOA* are downregulated in Alzheimer's disease [92, 93, 94]. Progenitor cells in the cerebral cortex have high levels of *ALDOA* and a change in energy metabolism associated with altered 16p11.2 gene dosage could impact neurogenesis, neuronal differentiation, and brain development [94]. Knockdown of the zebrafish homolog also resulted in defective brain and tail morphology [43]. These findings could explain ALDOA association with the 16pdel symptoms of psychiatric disorders, epilepsy or seizures, ASD, obesity and dysmorphic features.

## 4 Hypothesis: Synthesis and Promise

### 4.1 Disrupted Metabolic Pathways: Key Players in 16pdel Phenotypes?

**FIGURE 5:**
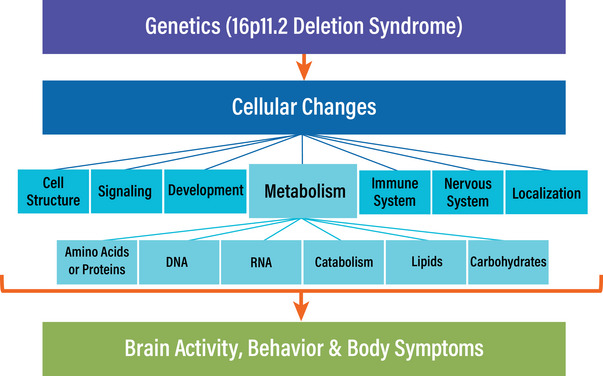
Model for contributions of 16p11.2 locus genes to 16p11.2 deletion syndrome, including changes in brain activity, behavior, and body. Changes in gene dosage in the 16p11.2 locus contribute to several cellular changes affecting specific functional categories, with some condensed for simplicity. These categories are cell structure (cellular process, response to stimulus), signaling, development (reproduction, homeostasis), metabolism, immune system, nervous system, and (establishment of) localization. Dysfunction in multiple metabolic processes (amino acid or proteins, DNA, RNA, catabolism, lipids, and carbohydrates) may be key contributors to 16p11.2 deletion syndrome.

We present the hypothesis that metabolic disruption after gene dosage changes is associated with multiple 16pdel phenotypes. This hypothesis arises from analyses that link phenotypes found in 16pdel with protein metabolism, DNA metabolism, RNA metabolism, catabolic processes, lipid metabolism, and carbohydrate metabolism (Figures 4 and 5, Table S4).

The data indicate that all 16pdel symptoms are under multigenic and combinatorial control of 16p11.2 genes (Figures 2 and 3). While for each set of 16pdel symptoms, both metabolic and non‐metabolic genes have been linked to symptomatology (Figure 2), in some cases a 16pdel symptom could primarily driven by changes in 16p11.2 metabolic gene dosage, while alternately, 16p11.2 metabolic and “non‐metabolic” genes may work together to drive other 16pdel symptoms. In support of our hypothesis, in Section 3.1, we explored how activities of specific 16p11.2 metabolic genes may contribute to aspects of 16pdel symptomatology. Table S4 aggregates the extensive data presented in Figures 2, 3, 4 to connect genes, symptomatology, combinatorial activity, and biological processes.

### 4.2 Primary Metabolic Control of 16pdel Symptoms

Which 16pdel symptoms might be under primary control of metabolic changes? In the aggregated data from Figures 2, 3, 4 and shown in Table S4, we conclude first, that all symptoms have at least one metabolic gene associated, as we define 10 out of the 27 16p11.2 genes to be direct metabolic regulators. Symptoms connected with a large number of metabolic genes may be targets for metabolic control. This may be the case with obesity (∼43% metabolic genes), ASD (40% metabolic genes), dysmorphic features (40% metabolic genes), and psychiatric disorders (∼34% metabolic genes), whereas metabolic genes likely play a lesser role in epilepsy or seizures (33% metabolic genes), cognitive impairment (∼29% metabolic genes), and language or motor delay (20% metabolic genes).

Second, many metabolic genes interact with another metabolic genes. For example, the synthesis of ceramides which control nervous system growth and support axon myelination [95], is regulated by TLCD3B, whereas CDIPT and GDPD3 synthesize the intermediate metabolites that regulate sphingolipid synthesis, including ceramides [82, 87, 90]. Thus, these three metabolic genes and their pathways may interact to regulate 16pdel symptoms. MAPK3 and TAOK2 are both members of the MAPK signaling pathway [65] and interaction may coordinately regulate pathways needed for neural development, potentially leading to brain signaling and structural changes [96]. Glucose metabolism plays an important role in providing the energy to accomplish metabolic activities [97]. *ALDOA* is involved in the energy‐producing process of glycolysis, where *MAPK3* also plays a role. Coordinate loss of function of both genes may compound dysregulation of energy homeostasis, associated with 16pdel symptoms.

### 4.3 Interactions of Metabolic and Non‐Metabolic Genes That May Control 16pdel Symptoms

Many non‐metabolic 16p11.2 genes are also associated with 16pdel phenotypes (Figure 2), and may interact with metabolic genes (Figure 3) to govern some 16pdel symptoms. An example of metabolic and non‐metabolic gene interaction is between the ceramide synthase modulator TLCD3B (metabolic) and the neurotransmitter release regulator DOC2A [55] (non‐metabolic) which are both associated with ASD (Figure 2). As noted in Section 2.2.2, TLCD3B and DOC2A interact in zebrafish to regulate seizure propensity [5] and coordinate loss of function could exacerbate 16pdel symptoms. Interactions between *MAPK3* and *TAOK2* (both metabolic genes) and MVP (non‐metabolic), which modulates the ERK signaling pathway, including *MAPK3* and *TAOK2*, may increase severity of 16pdel symptoms.

### 4.4 Explaining 16pdel Findings: Is Metabolic Dysfunction Causal?

Can the hypothesis of metabolic control as key to 16pdel be used to explain some specific findings in 16pdel mouse and iPSC models? One finding is that 16p11.2 deletion lengthens the cell cycle of ventral progenitors that is associated with premature differentiation into interneurons in organoids [98]. It is possible that this results from disruption of the MAPK signaling pathway and involves the 16p11.2 metabolic genes MAPK3 and TAOK2, possibly with contribution from non‐metabolic genes such as MVP. A second finding in a 16pdel model is altered neuronal morphology [47], number and frequency of neurons destined to populate the cortical lamina [71], both of which may also be affected by the MAPK signaling pathway. A third finding is the accelerated maturation of CA1 neurons in 16pdel mice, and the excitatory/inhibitory balance titled in favor of excitation [99]. This could be explained by loss of excitatory/inhibitory balance that is expected from QPRT loss of function due to the build‐up of the excitatory quinolinate.

### 4.5 Therapeutic Metabolic Interventions: A Promising Future for 16pdel Syndrome Treatment?

16pdel cannot presently be reversed, although some symptoms can be managed with medication, for example, anti‐seizure medications for seizures [5] or the γ‐aminobutyric acid (GABA) receptor agonist R‐baclofen for cognitive and social deficits [100]. The hypothesis that metabolic pathway changes or disruptions contribute to the symptomatology of 16pdel suggests that interventions targeting an affected pathway may be avenues for future treatments. Significant research is required to reach the goal of metabolic interventions, since current limitations due to small sample sizes or complex mechanisms of action make precise metabolic changes observed in 16pdel cells unclear [48, 101]. Nonetheless, there are several approaches at present that can be explored.

#### 4.5.1 Drugs That May Target 16pdel Metabolic Pathways

In consideration of interventions that may assist people affected with 16pdel syndrome, we probed several FDA‐approved and other potential drugs that act on the same pathways as the 16pdel metabolic genes (Table S3). We speculate that these could ameliorate symptoms associated with specific 16pdel metabolic genes (Section 3.1), and those identified as interacting with these genes (Figure 3, Table S4).

With regard to protein metabolism, drugs that could be useful in ameliorating disruption of the MAPK signaling pathway include Doxorubicin and Docetaxel which activate the pathway. With regard to DNA metabolism, Nicotinamide Riboside is an example of a drug that could be useful in ameliorating the disruption to DNA repair due to the loss of function of *PPP4C* and *MAPK3*. With regard to RNA metabolism, raloxifene could be useful in ameliorating a disruption to transcription due to *MAZ* and *MAPK3* loss of function as it is a selective estrogen receptor modulator that can activate the estrogen signaling pathway, which can promote transcription. With regard to the catabolic process class, drugs that could be useful in ameliorating the buildup of quinolinate due to QPRT loss of function include Epacadostat and LM10 which inhibit the quinolinate synthetic pathway, Nicotinamide Riboside, which is a precursor to a quinolinate degradation product, which could be in short supply if quinolinate degradation is reduced and Clofibrate, which can activate QPRT. With regard to lipid metabolism, drugs that could ameliorate changes to ceramide levels due to the loss of function of *TLCD3B* include Citicoline, a ceramide derivative analog, or N‐oleoyl‐ethanolamine, an inhibitor of ceramide degradation. Drugs that could be useful in ameliorating the changes to phospholipid levels include SC79 and SF1670 which promote the activity of PI3K (EC 2.7.1.137), which acts as a signal transducer by generating phospholipids [102] derived from the CDIPT phosphatidylinositol pathway. With regard to carbohydrate metabolism, Terazosin and Doxazosin are glycolytic enhancing drugs that may ameliorate disruption of glycolysis due to *ALDOA* loss of function.

#### 4.5.2 Other Therapeutic Interventions That May Target 16pdel Metabolic Dysfunction

Other useful metabolic 16pdel interventions outside of known drugs may include dietary or gene therapy approaches. For example, Omega‐3 polyunsaturated fatty acid supplementation can reduce the synthesis of and lower the concentration of ceramides in plasma and adipose tissues, which may help to overcome the effects of *TLCD3B* loss of function [103]. The ketogenic diet (high fat, low carbohydrate) also affects ceramides, with an increase in liver ceramide levels and synthesis of beneficial very‐long chain ceramides [104]. This diet also reduces diacylglycerol levels [104], intermediates in the phosphatidylinositol cycle [82, 87] and could help restore the imbalance after *CDIPT*, *TLCD3B*, and *GDPD3* loss of function.

Gene therapy aims to restore the normal function of a defective gene or to replace a mutant gene [105]. This can be accomplished using gene editing platforms such as the CRISPR/Cas system, viral gene delivery vehicles such as adenoviruses or non‐viral delivery systems such as mRNA [105]. For example, in the rat model temporal lobe epilepsy can be ameliorated through overexpression of the neural transcription factor NeuroD1 using an adeno‐associated virus vector, resulting in regeneration of GABAergic neurons in rats [106]. CRISPR interference has been used to block the function of *Fabp4*, which has the effect of reducing body weight in mice [107]. The challenges of gene therapy for 16pdel amelioration are that multiple genes would likely need to be simultaneously targeted, and gene therapy can presently affect only limited number of cells in any system.

## 5 Conclusion: Deciphering the Complexity of 16pdel

The 16p11.2 copy number variant locus can be deleted or duplicated, and when this occurs, it is tightly associated with myriad severe symptoms [19, 21]. In our analysis of gene‐symptom associations in 16pdel, several conclusions lead to a clarified model (Figure 5). In this, associated cellular changes connect with specific biological pathways and targets, including metabolism, transcriptional regulation, cell structure, neurotransmission, DNA repair, cell signaling, cell cycle, apoptosis, and the immune system. Due to the density of metabolic genes in the locus, we hypothesize that metabolic alterations are key contributors to 16pdel.

Our conclusions are three‐fold. First, genes within the 16p11.2 deletion interval are versatile such that many show associations with one or more 16pdel symptoms (Figure 2). Second, most 16p11.2 genes show functional interactions, indicating complex connectivity and synergy for the suite of 16pdel symptoms (Figure 3). Third, many of the 16p11.2 genes are directly or indirectly associated with metabolic regulation. Some of these are enzymes or direct enzyme modulators, others would likely act indirectly on cellular biochemistry (Figure 4). We draw the hypothesis that metabolic gene function in the 16p11.2 region contributes to 16pdel through perturbation of amino acid or protein, DNA and RNA, catabolism, lipid and energy (carbohydrate) metabolic pathways, arising from changes in gene copy number and/or interruption of coordinated or synergistic interactions.

We conclude that a productive goal for the field is to examine metabolic pathways in 16pdel, using animal models, human iPSC and organoids, as well as computational analysis. Identifying targeted, helpful treatments based on existing and new studies will improve the outcomes for people affected by 16p11.2 deletion syndrome.

### Author Contributions

Brandon Kar Meng Choo: Conceptualization, Writing ‐ OriginalDraft, Visualization. Sarah Barnes: Conceptualization, Writing ‐ OriginalDraft, Visualization. Hazel Sive: Conceptualization, Writing ‐ Review & Editing, Supervision, Funding acquisition.

## Conflicts of Interest

The authors declare that there are no conflicts of interest.

## Supporting information



Supporting Information

## Data Availability

Data sharing are not applicable to this article as no new data were created or analyzed in this study.
