# Supplementary material for: A Hypothesis: Metabolic Contributions to 16p11.2 Deletion Syndrome
Source: Bioessays. 2024 Dec 29;47(3):e202400177. doi: 10.1002/bies.202400177 (PMC11848116; doi:10.1002/bies.202400177)
Supplement: Supplementary file 1 — Supporting Information [file BIES-47-e202400177-s001.docx]

**Table S1.** Single gene variant-phenotype associations identified by Genome-Wide Association Studies or Whole Exome Sequencing.

| **16p11.2 Gene** | **Associated Phenotype** | **Reference** |
| --- | --- | --- |
| *TAOK2* | Autism Spectrum Disorder | ^[38]^ |
| *CORO1A* | Severe Combined Immunodeficiency | ^[20]^ |
| *PRRT2* | Epilepsy or Seizures | ^[16,23,51]^ |
| *TBX6* | Dysmorphic Features | ^[43,49,52]^ |
| *TLCD3B* | Autism Spectrum Disorder | ^[39]^ |

**Table S2.** 16pdel genes and homologs that have been studied in experimental systems for associations to 16pdel-like phenotypes. Haploinsufficiency: genetic loss of one functional gene copy; knockdown: using antisense RNA or oligonucleotides; knockout: genetic loss of both functional gene copies; chemical inhibition: inhibits enzyme function to mimic genetic loss of function.

| **16p11.2 Gene** | **Experimental Method to Target Gene** | **Experimental Tool and Reference** | | | | |
| --- | --- | --- | --- | --- | --- | --- |
|  |  | Cells | Flies | Mice | Rats | Zebrafish |
| *SPN* | Haploinsufficiency |  |  |  |  | ^[37]^ |
| *QPRT* | Haploinsufficiency, Knockdown, Knockout, Chemical Inhibition | ^[14,54]^ |  |  |  |  |
| *C16orf54* | Haploinsufficiency |  |  |  |  | ^[37]^ |
| *KIF22* | Knockdown |  | ^[33]^ |  |  | ^[4]^ |
| *MAZ* | Haploinsufficiency, Knockdown, Knockout | ^[13]^ |  | ^[13,31]^ |  | ^[4]^ |
| *PAGR1* | Knockdown |  |  |  |  | ^[4]^ |
| *MVP* | Haploinsufficiency, Knockdown | ^[3]^ |  | ^[3]^ |  | ^[4]^ |
| *CDIPT* | Haploinsufficiency, Knockdown |  | ^[17]^ |  |  | ^[4,32]^ |
| *SEZ6L2* | Haploinsufficiency, Knockdown |  |  |  |  | ^[4,37]^ |
| *ASPHD1* | Haploinsufficiency, Knockdown |  |  |  |  | ^[4,37]^ |
| *KCTD13* | Haploinsufficiency, Knockdown, Knockout | ^[21]^ | ^[17]^ | ^[2,8,10-11,29]^ | ^[28]^ | ^[4,10,37]^ |
| *TAOK2* | Haploinsufficiency, Knockdown, Knockout | ^[38]^ | ^[1]^ | ^[38]^ |  | ^[4,37]^ |
| *HIRIP3* | Knockdown |  |  |  |  | ^[4]^ |
| *INO80E* | Haploinsufficiency, Knockdown |  |  |  |  | ^[4,37]^ |
| *DOC2A* | Knockdown, Knockout, Epileptic Rats |  | ^[17]^ | ^[45]^ | ^[15]^ | ^[4]^ |
| *TLCD3B* | Haploinsufficiency, Knockdown, Knockout, High Fat Diet |  | ^[17]^ | ^[50]^ |  | ^[4,30,37]^ |
| *ALDOA* | Knockdown |  |  |  |  | ^[4]^ |
| *PPP4C* | Knockdown |  | ^[17,55]^ |  |  | ^[4]^ |
| *TBX6* | Haploinsufficiency, Knockdown, Knockout |  |  | ^[52-53]^ |  | ^[4]^ |
| *YPEL3* | Knockdown |  |  |  |  | ^[4]^ |
| *GDPD3* | Knockdown |  |  |  |  | ^[4]^ |
| *MAPK3* | Haploinsufficiency, Knockdown, Knockout |  | ^[17,34]^ | ^[19,35]^ | ^[28,36]^ | ^[4]^ |
| *CORO1A* | Knockdown |  |  |  |  | ^[4]^ |

**Table S3.** Drugs that may possibly affect the metabolic pathways that metabolic 16pdel genes are involved in.

| Pathway | Drug | Mechanism | Reference |
| --- | --- | --- | --- |
| Amino Acids or Proteins | Doxorubicin | Activates the MAPK signaling pathway | ^[26]^ |
|  | Paclitaxel | Activates the MAPK signaling pathway | ^[26]^ |
|  | Docetaxel | Activates the MAPK signaling pathway | ^[41]^ |
|  | Vemurafenib | Activates the MAPK signaling pathway in cells with wild-type RAF | ^[5]^ |
|  | Dabrafenib | Activates the MAPK signaling pathway in cells with wild-type RAF | ^[5]^ |
| DNA | Nicotinamide Riboside | A NAD+ precursor which is a substrate for PARP DNS repair enzymes | ^[48]^ |
| RNA | Raloxifene | Activates the estrogen signaling pathway which promotes transcription | ^[7]^ |
| Catabolism (Quinolinate) | Epacadostat | Inhibits IDO1, which participates in the first step of the Kynurenine Pathway. | ^[6]^ |
|  | Navoximod | Inhibits IDO1, which participates in the first step of the Kynurenine Pathway. | ^[6]^ |
|  | BMS-986205 | Inhibits IDO1, which participates in the first step of the Kynurenine Pathway. | ^[6]^ |
|  | LM10 | Inhibits TDO, which participates in the first step of the Kynurenine Pathway. | ^[6]^ |
|  | 680C91 | Inhibits TDO, which participates in the first step of the Kynurenine Pathway. | ^[6]^ |
|  | Nicotinamide Riboside | A NAD+ precursor which can potentially counteract a reduction in NAD+ levels due to a lack of quinolinate degradation | ^[6]^ |
|  | Clofibrate | QPRT agonist | ^[46]^ |
| Lipids (Ceramides) | Citicoline | Citicoline is broken down into choline in the body to potentially counteract a reduction in choline levels due to a lack of lysophosphatidylcholine degradation to form choline. | ^[42,47]^ |
|  | Fingolimod (FTY720) | Structural analogue of sphingosine | ^[12]^ |
|  | Synthetic Short/Long Chain Ceramide Analogs | Synthetic Ceramide Analog | ^[44]^ |
|  | N-oleoyl-ethanolamine (NOE) | Ceramidase Inhibitor | ^[44]^ |
|  | SKI-II (Sphingosine Kinase Inhibitor II) | Inhibits sphingosine kinase which forms S1P, decreasing the S1P/ceramide ratio and thereby promotes ceramide action | ^[44]^ |
| Lipids (Phospholipids) | SC79 | Activates AKT, which is a downstream target of PI3K | ^[18]^ |
|  | 740 Y-P | Agonist of PI3K | ^[24]^ |
|  | SF1670 | Inhibits PTEN, a negative regulator of PI3K | ^[9]^ |
|  | VO-OHpic | Inhibits PTEN, a negative regulator of PI3K | ^[27]^ |
| Carbohydrates  (Glycolysis) | Terazosin | Activates PGK1 to enhance glycolysis | ^[40]^ |
|  | Doxazosin | Activates PGK1 to enhance glycolysis | ^[40]^ |
|  | Alfuzosin | Activates PGK1 to enhance glycolysis | ^[40]^ |

IDO1: Indoleamine 2, 3-dioxygenase 1; TDO: Tryptophan-2,3-dioxygenase; NAD: Nicotinamide adenine dinucleotide; S1P: Sphingosine-1-phosphate; AKT: Protein Kinase B; PARP: Poly (ADP-ribose) Polymerase; PI3K: Phosphoinositide 3-kinase; PTEN: Phosphatase and Tensin homolog; PGK1: Phosphoglycerate Kinase 1; RAF: Rapidly Accelerated Fibrosarcoma.

**Table S4.** The **metabolic** and non-metabolic genes associated with common 16pdel symptoms, which other 16p11.2 genes they interact with, as well as the processes mapped to each gene.

| Phenotype | Associated **Metabolic** and Non-Metabolic Genes | **Metabolic** and Non-Metabolic Gene Interactions | Mapped **Metabolic** and Non-Metabolic Processes |
| --- | --- | --- | --- |
| Cognitive Impairment | SPN | - | - |
|  | PRRT2 | - | - Cellular Processes - Signaling - Nervous System   Establishment of Localization |
|  | SEZ6L2 | - **TAOK2**^[30]^ - **GDPD3**^[30]^ | - |
|  | INO80E | - KIF22^[30]^ - **ASPHD1**^[30]^ | - Response to Stimulus - Cellular Processes - Developmental - **DNA Metabolism** - **RNA Metabolism** |
|  | DOC2A | - KIF22^[30]^ - **CDIPT**^[17]^ - **TLCD3B**^[30]^ - **ALDOA**^[17]^ - **PPP4C**^[17]^ | - Signaling - Developmental   Establishment of Localization |
|  | **ALDOA** | - KCTD13^[17]^ - DOC2A^[17]^ - **MAPK3**^[17]^ | - Cellular Processes - Developmental - Homeostatic - Reproductive - **Catabolic Process** - **Carbohydrate Metabolism** |
|  | **MAPK3** | - KIF22^[25]^ - PAGR1^[17]^ - MVP^[22,37]^ - **CDIPT**^[17]^ - KCTD13^[10,37]^ - **TAOK2**^[25]^ - **TLCD3B**^[17]^ - **ALDOA**^[17]^ - **PPP4C**^[25]^ - TBX6^[17]^ - YPEL3^[17]^ - CORO1A^[17]^ | - Response to Stimulus - Cellular Processes - Signaling - Developmental - Immune System - Nervous System - Establishment of Localization - Homeostatic - Reproductive - **Protein Metabolism** - **DNA Metabolism** - **RNA Metabolism** - **Catabolic Process** |
| Psychiatric Disorders | MVP | - KCTD13^[10,37]^ - **MAPK3**^[22,37]^ | - Response to Stimulus - Signaling - Establishment of Localization - **Protein Metabolism** |
|  | **ALDOA** | - KCTD13^[17]^ - DOC2A^[17]^ - **MAPK3**^[17]^ | - Cellular Processes - Developmental - Homeostatic - Reproductive - **Catabolic Process** - **Carbohydrate Metabolism** |
|  | PRRT2 | - | - Cellular Processes - Signaling - Nervous System - Establishment of Localization |
|  | INO80E | - KIF22^[30]^ - **ASPHD1**^[30]^ | - Response to Stimulus - Cellular Processes - Developmental - **DNA Metabolism** - **RNA Metabolism** |
|  | **MAPK3** | - KIF22^[25]^ - PAGR1^[17]^ - MVP^[22,37]^ - **CDIPT**^[17]^ - KCTD13^[10,37]^ - **TAOK2**^[25]^ - **TLCD3B**^[17]^ - **ALDOA**^[17]^ - **PPP4C**^[25]^ - TBX6^[17]^ - YPEL3^[17]^ - CORO1A^[17]^ | - Response to Stimulus - Cellular Processes - Signaling - Developmental - Immune System - Nervous System - Establishment of Localization - Homeostatic - **Protein Metabolism** - **DNA Metabolism** - **RNA Metabolism** - **Catabolic Process** |
|  | CORO1A | - KCTD13^[17]^ - **PPP4C**^[17]^ - **MAPK3**^[17]^ | - Response to Stimulus - Cellular Processes - Signaling - Developmental - Immune System - Establishment of Localization - Homeostatic |
| Obesity | **QPRT** | - | - **Catabolic Process** |
|  | PRRT2 | - | - Cellular Processes - Signaling - Nervous System - Establishment of Localization |
|  | MVP | - KCTD13^[10,37]^ - **MAPK3**^[22,37]^ | - Response to Stimulus - Signaling - Establishment of Localization - **Protein Metabolism** |
|  | KCTD13 | - PAGR1^[17]^ - MVP^[10,37]^ - **CDIPT**^[17,30]^ - **TLCD3B**^[17,30]^ - **ALDOA**^[17]^ - **PPP4C**^[30]^ - YPEL3^[17]^ - **MAPK3**^[10,37]^ - CORO1A^[17]^ | - Response to Stimulus - Cellular Processes - Signaling - **Protein Metabolism** - **DNA Metabolism** - **Catabolic Process** |
|  | INO80E | - KIF22^[30]^ - **ASPHD1**^[30]^ | - Response to Stimulus - Cellular Processes - Developmental - **DNA Metabolism** - **RNA Metabolism** |
|  | **ALDOA** | - KCTD13^[17]^ - DOC2A^[17]^ - **MAPK3**^[17]^ | - Cellular Processes - Developmental - Homeostatic - Reproductive - **Catabolic Process** - **Carbohydrate Metabolism** |
|  | **MAPK3** | - KIF22^[25]^ - PAGR1^[17]^ - MVP^[22,37]^ - **CDIPT**^[17]^ - KCTD13^[10,37]^ - **TAOK2**^[25]^ - **TLCD3B**^[17]^ - **ALDOA**^[17]^ - **PPP4C**^[25]^ - TBX6^[17]^ - YPEL3^[17]^ - CORO1A^[17]^ | - Response to Stimulus - Cellular Processes - Signaling - Developmental - Immune System - Nervous System - Establishment of Localization - Homeostatic - **Protein Metabolism** - **DNA Metabolism** - **RNA Metabolism** - **Catabolic Process** |
| Language and Motor Delay | PRRT2 | - | - Cellular Processes - Signaling - Nervous System - Establishment of Localization |
|  | SEZ6L2 | - **TAOK2**^[30]^ - **GDPD3**^[30]^ | - |
|  | KCTD13 | - PAGR1^[17]^ - MVP^[10,37]^ - **CDIPT**^[17,30]^ - **TLCD3B**^[17,30]^ - **ALDOA**^[17]^ - **PPP4C**^[30]^ - YPEL3^[17]^ - **MAPK3**^[10,37]^ - CORO1A^[17]^ | - Response to Stimulus - Cellular Processes - Signaling - **Protein Metabolism** - **DNA Metabolism** - **Catabolic Process** |
|  | DOC2A | - KIF22^[30]^ - **CDIPT**^[17]^ - **TLCD3B**^[30]^ - **ALDOA**^[17]^ - **PPP4C**^[17]^ | - Signaling - Developmental - Establishment of Localization |
|  | **MAPK3** | - KIF22^[25]^ - PAGR1^[17]^ - MVP^[22,37]^ - **CDIPT**^[17]^ - KCTD13^[10,37]^ - **TAOK2**^[25]^ - **TLCD3B**^[17]^ - **ALDOA**^[17]^ - **PPP4C**^[25]^ - TBX6^[17]^ - YPEL3^[17]^ - CORO1A^[17]^ | - Response to Stimulus - Cellular Processes - Signaling - Developmental - Immune System - Nervous System - Establishment of Localization - Homeostatic - **Protein Metabolism** - **DNA Metabolism** - **RNA Metabolism** - **Catabolic Process** |
| Autism Spectrum Disorder | **QPRT** | - | - **Catabolic Process** |
|  | C16orf54 | - | - |
|  | KIF22 | - **TAOK2**^[30]^ - INO80E^[30]^ - HIRIP3^[30]^ - DOC2A^[30]^ - **MAPK3**^[25]^ | - Response to Stimulus - Cellular Processes - Establishment of Localization - **DNA Metabolism** |
|  | PRRT2 | - | - Cellular Processes - Signaling - Nervous System - Establishment of Localization |
|  | PAGR1 | - KCTD13^[17]^ - **PPP4C**^[17]^ - **MAPK3**^[17]^ | - Response to Stimulus - Signaling - **RNA Metabolism** |
|  | MVP | - KCTD13^[10,37]^ - **MAPK3**^[22,37]^ | - Response to Stimulus - Signaling - Establishment of Localization - **Protein Metabolism** |
|  | **CDIPT** | - KCTD13^[17,30]^ - **PPP4C**^[17]^ - DOC2A^[17]^ - **MAPK3**^[17]^ | - **Lipid Metabolism** |
|  | SEZ6L2 | - **TAOK2**^[30]^ - **GDPD3**^[30]^ | - |
|  | **ASPHD1** | - HIRIP3^[30]^ - INO80E^[30]^ - **PPP4C**^[30]^ | - **Protein Metabolism** |
|  | KCTD13 | - PAGR1^[17]^ - MVP^[10,37]^ - **CDIPT**^[17,30]^ - **TLCD3B**^[17,30]^ - **ALDOA**^[17]^ - **PPP4C**^[30]^ - YPEL3^[17]^ - **MAPK3**^[10,37]^ - CORO1A^[17]^ | - Response to Stimulus - Cellular Processes - Signaling - **Protein Metabolism** - **DNA Metabolism** - **Catabolic Process** |
|  | **TAOK2** | - KIF22^[30]^ - SEZ6L2^[30]^ - **MAPK3**^[25]^ | - Response to Stimulus - Cellular Processes - Signaling - Developmental - Establishment of Localization - **Protein Metabolism** |
|  | HIRIP3 | - KIF22^[30]^ - **ASPHD1**^[30]^ - **TLCD3B**^[30]^ | - Cellular Processes |
|  | INO80E | - KIF22^[30]^ - **ASPHD1**^[30]^ | - Response to Stimulus - Cellular Processes - Developmental - **DNA Metabolism** - **RNA Metabolism** |
|  | DOC2A | - KIF22^[30]^ - **CDIPT**^[17]^ - **TLCD3B**^[30]^ - **ALDOA**^[17]^ - **PPP4C**^[17]^ | - Signaling - Developmental - Establishment of Localization |
|  | C16orf92 | - | - Cellular Processes - Reproductive |
|  | **TLCD3B** | - KCTD13^[17,30]^ - HIRIP3^[30]^ - DOC2A^[17,30]^ - **ALDOA**^[30]^ - **MAPK3**^[17]^ | - Developmental - Homeostatic - **Lipid Metabolism** |
|  | **ALDOA** | - KCTD13^[17]^ - DOC2A^[17]^ - **MAPK3**^[17]^ | - Cellular Processes - Developmental - Homeostatic - Reproductive - **Catabolic Process** - **Carbohydrate Metabolism** |
|  | **PPP4C** | - PAGR1^[17]^ - **CDIPT**^[17]^ - **ASPHD1**^[30]^ - KCTD13^[30]^ - DOC2A^[17]^ - YPEL3^[17]^ - **MAPK3**^[25]^ - CORO1A^[17]^ | - Response to Stimulus - Signaling - **Protein Metabolism** - **DNA Metabolism** |
|  | TBX6 | - **MAPK3**^[17]^ | - Response to Stimulus - Cellular Processes - Signaling - Developmental - **RNA Metabolism** |
|  | YPEL3 | - KCTD13^[17]^ - **PPP4C**^[17]^ - **MAPK3**^[17]^ | - Response to Stimulus |
|  | **GDPD3** | - SEZ6L2^[30]^ | - **Catabolic Process** |
|  | **MAPK3** | - KIF22^[25]^ - PAGR1^[17]^ - MVP^[22,37]^ - **CDIPT**^[17]^ - KCTD13^[10,37]^ - **TAOK2**^[25]^ - **TLCD3B**^[17]^ - **ALDOA**^[17]^ - **PPP4C**^[25]^ - TBX6^[17]^ - YPEL3^[17]^ - CORO1A^[17]^ | - Response to Stimulus - Cellular Processes - Signaling - Developmental - Immune System - Nervous System - Establishment of Localization - Homeostatic - **Protein Metabolism** - **DNA Metabolism** - **RNA Metabolism** - **Catabolic Process** |
|  | CORO1A | - KCTD13^[17]^ - **PPP4C**^[17]^ - **MAPK3**^[17]^ | - Response to Stimulus - Cellular Processes - Signaling - Developmental - Immune System - Establishment of Localization - Homeostatic |
| Dysmorphic Features | **QPRT** | - | - **Catabolic Process** |
|  | C16orf54 | - | - |
|  | ZG16 | - | - Response to Stimulus - Establishment of Localization |
|  | KIF22 | - **TAOK2**^[30]^ - INO80E^[30]^ - HIRIP3^[30]^ - DOC2A^[30]^ - **MAPK3**^[25]^ | - Response to Stimulus - Cellular Processes - Establishment of Localization - **DNA Metabolism** |
|  | **MAZ** | - | - Response to Stimulus - Cellular Processes - Signaling - **RNA Metabolism** |
|  | PRRT2 | - | - Cellular Processes - Signaling - Nervous System - Establishment of Localization |
|  | MVP | - KCTD13^[10,37]^ - **MAPK3**^[22,37]^ | - Response to Stimulus - Signaling - Establishment of Localization - **Protein Metabolism** |
|  | **CDIPT** | - KCTD13^[17,30]^ - **PPP4C**^[17]^ - DOC2A^[17]^ - **MAPK3**^[17]^ | - **Lipid Metabolism** |
|  | SEZ6L2 | - **TAOK2**^[30]^ - **GDPD3**^[30]^ | - |
|  | **ASPHD1** | - HIRIP3^[30]^ - INO80E^[30]^ - **PPP4C**^[30]^ | - **Protein Metabolism** |
|  | KCTD13 | - PAGR1^[17]^ - MVP^[10,37]^ - **CDIPT**^[17,30]^ - **TLCD3B**^[17,30]^ - **ALDOA**^[17]^ - **PPP4C**^[30]^ - YPEL3^[17]^ - **MAPK3**^[10,37]^ - CORO1A^[17]^ | - Response to Stimulus - Cellular Processes - Signaling - **Protein Metabolism** - **DNA Metabolism** - **Catabolic Process** |
|  | TMEM219 | - | - Cellular Processes |
|  | **TAOK2** | - KIF22^[30]^ - SEZ6L2^[30]^ - **MAPK3**^[25]^ | - Response to Stimulus - Cellular Processes - Signaling - Developmental - Establishment of Localization - **Protein Metabolism** |
|  | INO80E | - KIF22^[30]^ - **ASPHD1**^[30]^ | - Response to Stimulus - Cellular Processes - Developmental - **DNA Metabolism** - **RNA Metabolism** |
|  | **TLCD3B** | - KCTD13^[17,30]^ - HIRIP3^[30]^ - DOC2A^[17,30]^ - **ALDOA**^[30]^ - **MAPK3**^[17]^ | - Developmental - Homeostatic - **Lipid Metabolism** |
|  | **ALDOA** | - KCTD13^[17]^ - DOC2A^[17]^ - **MAPK3**^[17]^ | - Cellular Processes - Developmental - Homeostatic - Reproductive - **Catabolic Process** - **Carbohydrate Metabolism** |
|  | TBX6 | - **MAPK3**^[17]^ | - Response to Stimulus - Cellular Processes - Signaling - Developmental - **RNA Metabolism** |
|  | YPEL3 | - KCTD13^[17]^ - **PPP4C**^[17]^ - **MAPK3**^[17]^ | - Response to Stimulus |
|  | **MAPK3** | - KIF22^[25]^ - PAGR1^[17]^ - MVP^[22,37]^ - **CDIPT**^[17]^ - KCTD13^[10,37]^ - **TAOK2**^[25]^ - **TLCD3B**^[17]^ - **ALDOA**^[17]^ - **PPP4C**^[25]^ - TBX6^[17]^ - YPEL3^[17]^ - CORO1A^[17]^ | - Response to Stimulus - Cellular Processes - Signaling - Developmental - Immune System - Nervous System - Establishment of Localization - Homeostatic - **Protein Metabolism** - **DNA Metabolism** - **RNA Metabolism** - **Catabolic Process** |
|  | CORO1A | - KCTD13^[17]^ - **PPP4C**^[17]^ - **MAPK3**^[17]^ | - Response to Stimulus - Cellular Processes - Signaling - Developmental - Immune System - Establishment of Localization - Homeostatic |
| Epilepsy or Seizures | **QPRT** | - | - **Catabolic Process** |
|  | KIF22 | - **TAOK2**^[30]^ - INO80E^[30]^ - HIRIP3^[30]^ - DOC2A^[30]^ - **MAPK3**^[25]^ | - Response to Stimulus - Cellular Processes - Establishment of Localization - **DNA Metabolism** |
|  | PRRT2 | - | - Cellular Processes - Signaling - Nervous System - Establishment of Localization |
|  | MVP | - KCTD13^[10,37]^ - **MAPK3**^[22,37]^ | - Response to Stimulus - Signaling - Establishment of Localization - **Protein Metabolism** |
|  | SEZ6L2 | - **TAOK2**^[30]^ - **GDPD3**^[30]^ | - |
|  | **ASPHD1** | - HIRIP3^[30]^ - INO80E^[30]^ - **PPP4C**^[30]^ | - **Protein Metabolism** |
|  | KCTD13 | - PAGR1^[17]^ - MVP^[10,37]^ - **CDIPT**^[17,30]^ - **TLCD3B**^[17,30]^ - **ALDOA**^[17]^ - **PPP4C**^[30]^ - YPEL3^[17]^ - **MAPK3**^[10,37]^ - CORO1A^[17]^ | - Response to Stimulus - Cellular Processes - Signaling - **Protein Metabolism** - **DNA Metabolism** - **Catabolic Process** |
|  | INO80E | - KIF22^[30]^ - **ASPHD1**^[30]^ | - Response to Stimulus - Cellular Processes - Developmental - **DNA Metabolism** - **RNA Metabolism** |
|  | DOC2A | - KIF22^[30]^ - **CDIPT**^[17]^ - **TLCD3B**^[30]^ - **ALDOA**^[17]^ - **PPP4C**^[17]^ | - Signaling - Developmental - Establishment of Localization |
|  | **ALDOA** | - KCTD13^[17]^ - DOC2A^[17]^ - **MAPK3**^[17]^ | - Cellular Processes - Developmental - Homeostatic - Reproductive - **Catabolic Process** - **Carbohydrate Metabolism** |
|  | TBX6 | - **MAPK3**^[17]^ | - Response to Stimulus - Cellular Processes - Signaling - Developmental - **RNA Metabolism** |
|  | **MAPK3** | - KIF22^[25]^ - PAGR1^[17]^ - MVP^[22,37]^ - **CDIPT**^[17]^ - KCTD13^[10,37]^ - **TAOK2**^[25]^ - **TLCD3B**^[17]^ - **ALDOA**^[17]^ - **PPP4C**^[25]^ - TBX6^[17]^ - YPEL3^[17]^ - CORO1A^[17]^ | - Response to Stimulus - Cellular Processes - Signaling - Developmental - Immune System - Nervous System - Establishment of Localization - Homeostatic - **Protein Metabolism** - **DNA Metabolism** - **RNA Metabolism** - **Catabolic Process** |

**Supplementary References**

[1] Agrawal, N., Lawler, K., Davidson, C. M., Keogh, J. M., Legg, R., Interval, . . . Brand, A. H. (2021). Predicting novel candidate human obesity genes and their site of action by systematic functional screening in Drosophila. *PLoS Biol, 19*(11), e3001255. doi: 10.1371/journal.pbio.3001255

[2] Arbogast, T., Razaz, P., Ellegood, J., McKinstry, S. U., Erdin, S., Currall, B., . . . Katsanis, N. (2019). Kctd13-deficient mice display short-term memory impairment and sex-dependent genetic interactions. *Hum Mol Genet, 28*(9), 1474-1486. doi: 10.1093/hmg/ddy436

[3] Ben, J., Jiang, B., Wang, D., Liu, Q., Zhang, Y., Qi, Y., . . . Chen, Q. (2019). Major vault protein suppresses obesity and atherosclerosis through inhibiting IKK-NF-kappaB signaling mediated inflammation. *Nat Commun, 10*(1), 1801. doi: 10.1038/s41467-019-09588-x

[4] Blaker-Lee, A., Gupta, S., McCammon, J. M., De Rienzo, G., & Sive, H. (2012). Zebrafish homologs of genes within 16p11.2, a genomic region associated with brain disorders, are active during brain development, and include two deletion dosage sensor genes. *Dis Model Mech, 5*(6), 834-851. doi: 10.1242/dmm.009944

[5] Callahan, M. K., Rampal, R., Harding, J. J., Klimek, V. M., Chung, Y. R., Merghoub, T., . . . Chapman, P. B. (2012). Progression of RAS-mutant leukemia during RAF inhibitor treatment. *N Engl J Med, 367*(24), 2316-2321. doi: 10.1056/NEJMoa1208958

[6] Castro-Portuguez, R., & Sutphin, G. L. (2020). Kynurenine pathway, NAD(+) synthesis, and mitochondrial function: Targeting tryptophan metabolism to promote longevity and healthspan. *Exp Gerontol, 132*, 110841. doi: 10.1016/j.exger.2020.110841

[7] Engdahl, C., Jochems, C., Gustafsson, J. A., van der Saag, P. T., Carlsten, H., & Lagerquist, M. K. (2009). In vivo activation of gene transcription via oestrogen response elements by a raloxifene analogue. *J Endocrinol, 203*(3), 349-356. doi: 10.1677/joe-09-0012

[8] Escamilla, C. O., Filonova, I., Walker, A. K., Xuan, Z. X., Holehonnur, R., Espinosa, F., . . . Powell, C. M. (2017). Kctd13 deletion reduces synaptic transmission via increased RhoA. *Nature, 551*(7679), 227-231. doi: 10.1038/nature24470

[9] Fu, H. Y., Shen, L., Gao, X. S., Cui, D. X., & Cui, Z. Y. (2020). SF1670 inhibits apoptosis and inflammation via the PTEN/Akt pathway and thus protects intervertebral disc degeneration. *Eur Rev Med Pharmacol Sci, 24*(17), 8694-8702. doi: 10.26355/eurrev_202009_22806

[10] Golzio, C., Willer, J., Talkowski, M. E., Oh, E. C., Taniguchi, Y., Jacquemont, S., . . . Katsanis, N. (2012). KCTD13 is a major driver of mirrored neuroanatomical phenotypes of the 16p11.2 copy number variant. *Nature, 485*(7398), 363-367. doi: 10.1038/nature11091

[11] Gu, J., Ke, P., Guo, H., Liu, J., Liu, Y., Tian, X., . . . Xiao, F. (2023). KCTD13-mediated ubiquitination and degradation of GluN1 regulates excitatory synaptic transmission and seizure susceptibility. *Cell Death Differ, 30*(7), 1726-1741. doi: 10.1038/s41418-023-01174-5

[12] Guo, J., Feng, J., Qu, H., Xu, H., & Zhou, H. (2022). Potential Drug Targets for Ceramide Metabolism in Cardiovascular Disease. *J Cardiovasc Dev Dis, 9*(12). doi: 10.3390/jcdd9120434

[13] Haller, M., Au, J., O'Neill, M., & Lamb, D. J. (2018). 16p11.2 transcription factor MAZ is a dosage-sensitive regulator of genitourinary development. *Proc Natl Acad Sci U S A, 115*(8), E1849-E1858. doi: 10.1073/pnas.1716092115

[14] Haslinger, D., Waltes, R., Yousaf, A., Lindlar, S., Schneider, I., Lim, C. K., . . . Chiocchetti, A. G. (2018). Loss of the Chr16p11.2 ASD candidate gene QPRT leads to aberrant neuronal differentiation in the SH-SY5Y neuronal cell model. *Mol Autism, 9*(1), 56. doi: 10.1186/s13229-018-0239-z

[15] Hu, X., Tang, J., Lan, X., & Mi, X. (2019). Increased expression of DOC2A in human and rat temporal lobe epilepsy. *Epilepsy Res, 151*, 78-84. doi: 10.1016/j.eplepsyres.2019.02.008

[16] Huguet, G., Nava, C., Lemiere, N., Patin, E., Laval, G., Ey, E., . . . Bourgeron, T. (2014). Heterogeneous pattern of selective pressure for PRRT2 in human populations, but no association with autism spectrum disorders. *PLOS ONE, 9*(3), e88600. doi: 10.1371/journal.pone.0088600

[17] Iyer, J., Singh, M. D., Jensen, M., Patel, P., Pizzo, L., Huber, E., . . . Girirajan, S. (2018). Pervasive genetic interactions modulate neurodevelopmental defects of the autism-associated 16p11.2 deletion in Drosophila melanogaster. *Nat Commun, 9*(1), 2548. doi: 10.1038/s41467-018-04882-6

[18] Jo, H., Mondal, S., Tan, D., Nagata, E., Takizawa, S., Sharma, A. K., . . . Luo, H. R. (2012). Small molecule-induced cytosolic activation of protein kinase Akt rescues ischemia-elicited neuronal death. *Proc Natl Acad Sci U S A, 109*(26), 10581-10586. doi: 10.1073/pnas.1202810109

[19] Khan, A. S., Subramaniam, S., Dramane, G., Khelifi, D., & Khan, N. A. (2017). ERK1 and ERK2 activation modulates diet-induced obesity in mice. *Biochimie, 137*, 78-87. doi: 10.1016/j.biochi.2017.03.004

[20] Khoreva, A., Butov, K. R., Nikolaeva, E. I., Martyanov, A., Kulakovskaya, E., Pershin, D., . . . Shcherbina, A. (2024). Novel hemizygous *CORO1A* variant leads to combined immunodeficiency with defective platelet calcium signaling and cell mobility. *J Allergy Clin Immunol Glob, 3*(1), 100172. doi: 10.1016/j.jacig.2023.100172

[21] Kizner, V., Naujock, M., Fischer, S., Jager, S., Reich, S., Schlotthauer, I., . . . Gillardon, F. (2020). CRISPR/Cas9-mediated Knockout of the Neuropsychiatric Risk Gene KCTD13 Causes Developmental Deficits in Human Cortical Neurons Derived from Induced Pluripotent Stem Cells. *Mol Neurobiol, 57*(2), 616-634. doi: 10.1007/s12035-019-01727-1

[22] Kretz, P. F., Wagner, C., Mikhaleva, A., Montillot, C., Hugel, S., Morella, I., . . . Yalcin, B. (2023). Dissecting the autism-associated 16p11.2 locus identifies multiple drivers in neuroanatomical phenotypes and unveils a male-specific role for the major vault protein. *Genome Biol, 24*(1), 261. doi: 10.1186/s13059-023-03092-8

[23] Lee, J., Kim, Y. O., Lim, B. C., & Lee, J. (2023). PRRT2-positive self-limited infantile epilepsy: Initial seizure characteristics and response to sodium channel blockers. *Epilepsia Open, 8*(2), 436-443. doi: 10.1002/epi4.12708

[24] Li, D., Guo, Y. Y., Cen, X. F., Qiu, H. L., Chen, S., Zeng, X. F., . . . Tang, Q. Z. (2022). Lupeol protects against cardiac hypertrophy via TLR4-PI3K-Akt-NF-kappaB pathways. *Acta Pharmacol Sin, 43*(8), 1989-2002. doi: 10.1038/s41401-021-00820-3

[25] Liu, F., Liang, C., Li, Z., Zhao, S., Yuan, H., Yao, R., . . . Chen, X. (2023). Haplotype-specific MAPK3 expression in 16p11.2 deletion contributes to variable neurodevelopment. *Brain, 146*(8), 3347-3363. doi: 10.1093/brain/awad071

[26] Liu, J., Mao, W., Ding, B., & Liang, C. S. (2008). ERKs/p53 signal transduction pathway is involved in doxorubicin-induced apoptosis in H9c2 cells and cardiomyocytes. *Am J Physiol Heart Circ Physiol, 295*(5), H1956-1965. doi: 10.1152/ajpheart.00407.2008

[27] Mak, L. H., Vilar, R., & Woscholski, R. (2010). Characterisation of the PTEN inhibitor VO-OHpic. *J Chem Biol, 3*(4), 157-163. doi: 10.1007/s12154-010-0041-7

[28] Martin Lorenzo, S., Muniz Moreno, M. D. M., Atas, H., Pellen, M., Nalesso, V., Raffelsberger, W., . . . Herault, Y. (2023). Changes in social behavior with MAPK2 and KCTD13/CUL3 pathways alterations in two new outbred rat models for the 16p11.2 syndromes with autism spectrum disorders. *Front Neurosci, 17*, 1148683. doi: 10.3389/fnins.2023.1148683

[29] Martin Lorenzo, S., Nalesso, V., Chevalier, C., Birling, M. C., & Herault, Y. (2021). Targeting the RHOA pathway improves learning and memory in adult Kctd13 and 16p11.2 deletion mouse models. *Mol Autism, 12*(1), 1. doi: 10.1186/s13229-020-00405-7

[30] McCammon, J. M., Blaker-Lee, A., Chen, X., & Sive, H. (2017). The 16p11.2 homologs fam57ba and doc2a generate certain brain and body phenotypes. *Hum Mol Genet, 26*(19), 3699-3712. doi: 10.1093/hmg/ddx255

[31] Medina-Martinez, O., Haller, M., Rosenfeld, J. A., O'Neill, M. A., Lamb, D. J., & Jamrich, M. (2020). The transcription factor Maz is essential for normal eye development. *Dis Model Mech, 13*(8). doi: 10.1242/dmm.044412

[32] Murphy, T. R., Vihtelic, T. S., Ile, K. E., Watson, C. T., Willer, G. B., Gregg, R. G., . . . Hyde, D. R. (2011). Phosphatidylinositol synthase is required for lens structural integrity and photoreceptor cell survival in the zebrafish eye. *Exp Eye Res, 93*(4), 460-474. doi: 10.1016/j.exer.2011.06.010

[33] Park, S. M., Littleton, J. T., Park, H. R., & Lee, J. H. (2016). Drosophila Homolog of Human KIF22 at the Autism-Linked 16p11.2 Loci Influences Synaptic Connectivity at Larval Neuromuscular Junctions. *Experimental Neurobiology, 25*(1), 33-39. doi: 10.5607/en.2016.25.1.33

[34] Park, S. M., Park, H. R., & Lee, J. H. (2017). MAPK3 at the Autism-Linked Human 16p11.2 Locus Influences Precise Synaptic Target Selection at Drosophila Larval Neuromuscular Junctions. *Mol Cells, 40*(2), 151-161. doi: 10.14348/molcells.2017.2307

[35] Pucilowska, J., Vithayathil, J., Pagani, M., Kelly, C., Karlo, J. C., Robol, C., . . . Landreth, G. E. (2018). Pharmacological Inhibition of ERK Signaling Rescues Pathophysiology and Behavioral Phenotype Associated with 16p11.2 Chromosomal Deletion in Mice. *J Neurosci, 38*(30), 6640-6652. doi: 10.1523/JNEUROSCI.0515-17.2018

[36] Pucilowska, J., Vithayathil, J., Tavares, E. J., Kelly, C., Karlo, J. C., & Landreth, G. E. (2015). The 16p11.2 deletion mouse model of autism exhibits altered cortical progenitor proliferation and brain cytoarchitecture linked to the ERK MAPK pathway. *J Neurosci, 35*(7), 3190-3200. doi: 10.1523/JNEUROSCI.4864-13.2015

[37] Qiu, Y., Arbogast, T., Lorenzo, S. M., Li, H., Tang, S. C., Richardson, E., . . . Sebat, J. (2019). Oligogenic Effects of 16p11.2 Copy-Number Variation on Craniofacial Development. *Cell Rep, 28*(13), 3320-3328 e3324. doi: 10.1016/j.celrep.2019.08.071

[38] Richter, M., Murtaza, N., Scharrenberg, R., White, S. H., Johanns, O., Walker, S., . . . Calderon de Anda, F. (2019). Altered TAOK2 activity causes autism-related neurodevelopmental and cognitive abnormalities through RhoA signaling. *Mol Psychiatry, 24*(9), 1329-1350. doi: 10.1038/s41380-018-0025-5

[39] Satterstrom, F. K., Kosmicki, J. A., Wang, J., Breen, M. S., De Rubeis, S., An, J. Y., . . . Buxbaum, J. D. (2020). Large-Scale Exome Sequencing Study Implicates Both Developmental and Functional Changes in the Neurobiology of Autism. *Cell, 180*(3), 568-584 e523. doi: 10.1016/j.cell.2019.12.036

[40] Simmering, J. E., Welsh, M. J., Schultz, J., & Narayanan, N. S. (2022). Use of Glycolysis-Enhancing Drugs and Risk of Parkinson's Disease. *Mov Disord, 37*(11), 2210-2216. doi: 10.1002/mds.29184

[41] Su, C. C., Lin, J. W., Chang, K. Y., Wu, C. T., Liu, S. H., Chang, K. C., . . . Chen, Y. W. (2022). Involvement of AMPKalpha and MAPK-ERK/-JNK Signals in Docetaxel-Induced Human Tongue Squamous Cell Carcinoma Cell Apoptosis. *Int J Mol Sci, 23*(22). doi: 10.3390/ijms232213857

[42] Swiatkiewicz, M., & Grieb, P. (2023). Citicoline for Supporting Memory in Aging Humans. *Aging Dis, 14*(4), 1184-1195. doi: 10.14336/AD.2022.0913

[43] Tewes, A. C., Rall, K. K., Romer, T., Hucke, J., Kapczuk, K., Brucker, S., . . . Ledig, S. (2015). Variations in RBM8A and TBX6 are associated with disorders of the mullerian ducts. *Fertil Steril, 103*(5), 1313-1318. doi: 10.1016/j.fertnstert.2015.02.014

[44] Wajapeyee, N., Beamon, T. C., & Gupta, R. (2024). Roles and therapeutic targeting of ceramide metabolism in cancer. *Mol Metab, 83*, 101936. doi: 10.1016/j.molmet.2024.101936

[45] Wang, Q. W., Qin, J., Chen, Y. F., Tu, Y., Xing, Y. Y., Wang, Y., . . . Yao, J. (2023). 16p11.2 CNV gene Doc2alpha functions in neurodevelopment and social behaviors through interaction with Secretagogin. *Cell Rep, 42*(7), 112691. doi: 10.1016/j.celrep.2023.112691

[46] Wang, Z., Gao, Y., Zhang, C., Hu, H., Guo, D., Xu, Y., . . . Tang, H. (2017). Quinolinate Phosphoribosyltransferase is an Antiviral Host Factor Against Hepatitis C Virus Infection. *Sci Rep, 7*(1), 5876. doi: 10.1038/s41598-017-06254-4

[47] Wijayatunge, R., Holmstrom, S. R., Foley, S. B., Mgbemena, V. E., Bhargava, V., Perez, G. L., . . . Ross, T. S. (2018). Deficiency of the Endocytic Protein Hip1 Leads to Decreased Gdpd3 Expression, Low Phosphocholine, and Kypholordosis. *Mol Cell Biol, 38*(23). doi: 10.1128/MCB.00385-18

[48] Wilk, A., Hayat, F., Cunningham, R., Li, J., Garavaglia, S., Zamani, L., . . . Sobol, R. W. (2020). Extracellular NAD+ enhances PARP-dependent DNA repair capacity independently of CD73 activity. *Scientific Reports, 10*(1), 651. doi: 10.1038/s41598-020-57506-9

[49] Wu, N., Ming, X., Xiao, J., Wu, Z., Chen, X., Shinawi, M., . . . Zhang, F. (2015). TBX6 null variants and a common hypomorphic allele in congenital scoliosis. *N Engl J Med, 372*(4), 341-350. doi: 10.1056/NEJMoa1406829

[50] Yamashita-Sugahara, Y., Tokuzawa, Y., Nakachi, Y., Kanesaki-Yatsuka, Y., Matsumoto, M., Mizuno, Y., & Okazaki, Y. (2013). Fam57b (family with sequence similarity 57, member B), a novel peroxisome proliferator-activated receptor gamma target gene that regulates adipogenesis through ceramide synthesis. *J Biol Chem, 288*(7), 4522-4537. doi: 10.1074/jbc.M112.440792

[51] Yang, L., You, C., Qiu, S., Yang, X., Li, Y., Liu, F., . . . Li, B. (2020). Novel and de novo point and large microdeletion mutation in PRRT2-related epilepsy. *Brain Behav, 10*(5), e01597. doi: 10.1002/brb3.1597

[52] Yang, N., Wu, N., Dong, S., Zhang, L., Zhao, Y., Chen, W., . . . Zhang, F. (2020). Human and mouse studies establish TBX6 in Mendelian CAKUT and as a potential driver of kidney defects associated with the 16p11.2 microdeletion syndrome. *Kidney Int, 98*(4), 1020-1030. doi: 10.1016/j.kint.2020.04.045

[53] Yang, N., Wu, N., Zhang, L., Zhao, Y., Liu, J., Liang, X., . . . Zhang, F. (2019). TBX6 compound inheritance leads to congenital vertebral malformations in humans and mice. *Hum Mol Genet, 28*(4), 539-547. doi: 10.1093/hmg/ddy358

[54] Yang, Y., Booker, S. A., Clegg, J. M., Quintana-Urzainqui, I., Sumera, A., Kozic, Z., . . . Pratt, T. (2023). Identifying foetal forebrain interneurons as a target for monogenic autism risk factors and the polygenic 16p11.2 microdeletion. *BMC Neurosci, 24*(1), 5. doi: 10.1186/s12868-022-00771-3

[55] Yusuff, T., Jensen, M., Yennawar, S., Pizzo, L., Karthikeyan, S., Gould, D. J., . . . Girirajan, S. (2020). Drosophila models of pathogenic copy-number variant genes show global and non-neuronal defects during development. *PLoS Genet, 16*(6), e1008792. doi: 10.1371/journal.pgen.1008792
